# Biodiversity of freshwater planarians (Platyhelminthes, Tricladida, Dugesiidae) in Chile: exploration of unknown species

**DOI:** 10.1186/s12862-026-02501-3

**Published:** 2026-03-28

**Authors:** Andrés Lagos-Basoalto, Pamela Morales, Miguel L. Allende, Constanza Vásquez-Doorman

**Affiliations:** 1 Departamento de Biología, Facultad de Ciencias, Universidad de Chile, Santiago, Chile; 2 Escuela de Kinesiología & Departamento de Ciencias Químicas y Biológicas, Facultad de Salud, Universidad Bernardo O’Higgins, Santiago, Chile; 3 Millennium Institute Center for Genome Regulation, Santiago, Chile; 4 Departamento de Ecología y Biodiversidad, Facultad de Ciencias de la Vida, Universidad Andres Bello, Santiago, Chile

**Keywords:** Systematics, Biodiversity, Molecular species delimitation, Dugesiidae

## Abstract

**Background:**

Freshwater planarians (Tricladida) are widely used as model organisms in regeneration biology, invasion ecology, ecotoxicology, and systematics. Despite this relevance, their diversity remains poorly documented in large regions of South America. In Chile, only five freshwater planarian species have been formally described, a number that likely reflects limited sampling rather than true diversity. Given Chile’s pronounced latitudinal extent, biogeographic complexity, and environmental heterogeneity, freshwater planarian diversity is expected to be substantially underestimated. Here, we provide a first molecular-based assessment of Chilean freshwater planarians by sampling previously unexplored localities and integrating external morphology with phylogenetic and species delimitation analyses.

**Results:**

Specimens were collected from seven freshwater habitats spanning northern and central Chile, including high-altitude Altiplano environments and a geothermal field. External morphology was consistent with assignment to the genus *Girardia*, a conclusion supported by BLAST and BOLD searches. Phylogenetic analyses based on mitochondrial (COI) and nuclear (EF1-α) markers recovered three major Chilean clades with strong support, corresponding broadly to geographic regions. Altiplano populations formed a previously unreported monophyletic lineage within *Girardia*, suggesting isolation-driven diversification in extreme environments. Molecular species delimitation using ASAP, GMYC, and bPTP identified six putative species: *Girardia* sp. El Tatio, *Girardia* sp. Lauca/Chungará/Ascotán, *Girardia* sp. Calafquén, *Girardia* sp. OHP/LTB, *Girardia* sp. Los Lagos, and *Girardia* sp. LPM.

**Conclusion:**

Our results reveal that freshwater planarian diversity in Chile is far greater than currently recognized and that *Girardia* exhibits pronounced geographic structuring across the country. The discovery of multiple genetically distinct lineages, including in high-altitude and geothermal environments, highlights the role of geographic isolation and environmental heterogeneity in shaping diversification. Although formal species descriptions will require internal anatomy and additional molecular data, this study provides a critical framework for future taxonomic, evolutionary, and ecological research on South American freshwater planarians and underscores the importance of expanding biodiversity surveys into understudied regions.

**Supplementary Information:**

The online version contains supplementary material available at 10.1186/s12862-026-02501-3.

## Background

Freshwater planarians (Platyhelminthes, Tricladida) have characteristics that make them valuable for research in several areas, including regeneration biology, due to the activity of neoblasts, pluripotent adult stem cells capable of completely restoring any amputated body part [1, 2]; the ecology of invasive species, where literature increasingly reports the invasive behavior of planarians, demonstrating their remarkable ability to establish themselves in new environments, as they have proven to be formidable competitors in interspecific interactions, and in many ecosystems occupy the position of apex predators; in ecotoxicology, researchers use freshwater planarians as bioindicators, assessing the condition of urban rivers through genotoxicity studies [3, 4]; and systematics, especially with the advent of molecular phylogenies, which have enabled significant progress in the study of the relationships between members of the group, gradually elucidating unresolved branches of the tree of life ([5, 6(preprint)]).

Despite their relevance, species identification in Tricladida remains challenging. Flatworms have a simplified external morphology, which prevents species identification or delimitation due to the lack of distinctive features [7]. Most classifications rely on internal morphology, particularly the reproductive system, which has many distinguishing structures [8]. However, a molecular approach is an effective alternative and complement to traditional morphology/histology-based systematics. Molecular markers specific to Tricladida are used to study evolutionary relationships [9, 10], identify individuals through DNA barcoding [11, 12], and complement morphological studies for species delimitation [13, 14], among others.

Freshwater planarian biodiversity is relatively well documented in parts of Europe and Asia, as well as in some regions of Oceania ([6, 13, 15–20]). In contrast, knowledge remains scarce in large portions of South America. In Chile, for example, only five species of freshwater planarians have been described, four species belonging to the genus *Girardia *(Ball, 1974) [21] (*G. canai *(Curino & Cazzaniga, 1993) [22], *G. chilla *(Marcus, 1954) [23], *G. rincona *(Marcus, 1954) [23], *G. festai *(Borelli, 1898) [24]), and one of *Romankenkius *(Ball, 1974) [21] (*R. patagonicus *(Borelli, 1901) [25]) (summarized in [26]). This situation is similar to that in Peru and Colombia, with one and four species described, respectively, and much lower than in countries like Brazil and Argentina, which have eighteen and nine species, respectively, at the time of writing this manuscript (WoRMS Editorial Board, [27]). These data most likely reflect a sampling bias, as more analyses have been done in countries where scientists actively study this group.

Chile spans over 4,200 km from north to south, with diverse climates and biomes shaped by factors such as mountain ranges and ocean currents, creating microclimates within its regions [28]. Biogeographic isolation, such as the arid diagonal and interglacial cycles, has influenced the flora and fauna [29]. Given this, the reported diversity of freshwater planarians in Chile is likely severely underestimated. In this context, the present work aims to expand knowledge about Chilean freshwater planarians by collecting samples from previously unexplored areas, analyzing their external morphology, and conducting molecular phylogenetic and species delimitation studies, providing a first assessment of freshwater planarian diversity in the country.

## Materials and methods

### Sample collection

Specimens were collected from seven locations in northern and central ​​Chile (Table 1; Fig. 2A), between 2021 and 2025. Individuals were handpicked, stored in water from their environment in 50 ml centrifuge tubes, and transported to the laboratory. The exception was Calafquén Lake, where specimens were ethanol-fixed on site. Whenever possible, nearby rocks and stones were inspected for cocoons. Specimens were observed and photographed under a stereomicroscope (Motic SMZ-171) to evaluate taxonomically relevant traits, such as auricles and head shape, body and pharynx pigmentation, and presence of gonopores to assess whether or not individuals reproduce sexually. Individuals were starved for a week and preserved in absolute ethanol at -20 °C for molecular work. Additionally, genetic data available in GenBank from other localities were included in the study (these sites were not sampled in this study).

**Table 1:** Location of the samples collected. The name and location of the site, geographic coordinates, altitude, and number of specimens used for DNA extraction per area are shown

Location	Latitude	Longitude	Altitude (m.a.s.l.)	Sample ID	Number of DNA samples
Laucar River, Putre, Arica y Parinacota Region	-18,193889	-69,273889	4,490	ChiLauca	2
Chungará Lake, Putre, Arica y Parinacota Region	-18,235833	-69,181389	4,555	ChiChungará	2
Ascotán Salt Pan, Ollagüe, Antofagasta Region	-21,1978611	-68,2573889	3,725	ChiAscotán	2
El Tatio, Calama, Antofagasta Region	-22,375	-68,018056	4,335	ChiTatio	2
O’Higgins Park artificial pond, Santiago, Metropolitana Region	-33,4688611	-70,6609722	535	ChiOHP	1
Las Toscas Brook, Chillán, Ñuble Region	-36,600268	-72,0723557	130	ChiLTB	2
Calafquén Lake, Puerto Curihue, Los Ríos Region	-39,5445499	-72,1029345	205	ChiCalafquén	1

### Molecular methods

DNA was extracted from the ethanol-fixed individuals. For the Ascotán locality, each individual studied belonged to one of the two morphotypes. We followed the protocol by Leria et al., [30] with modifications. In summary, ethanol-preserved samples were rehydrated through a series of ethanol-to-PBSTx washes. Tissue samples were then incubated for 16 h at 37°C in a solution containing 190 µL of Lysis Buffer (Wizard^®^, Promega, Madison, WI, USA) or GTC buffer (4 M guanidinium thiocyanate, 25 mM citrate sodium, 0.5% w/v N-lauroylsarcosine, 7% v/v b-mercaptoethanol) and 10 µL of Proteinase K (20 mg/mL). Next, 12 µL of RNase A (10 mg/mL, Thermo Scientific) was added, followed by a 1-hour incubation at 37°C. DNA extraction was completed using the standard phenol-chloroform protocol [31]. DNA quality and quantity were assessed with a Nanodrop spectrophotometer (ThermoFisher). A fraction of the nuclear gene encoding *elongation factor 1-α* (EF1-α) and of the mitochondrial gene encoding *cytochrome oxidase I* (COI) were amplified by PCR, using the specific primers ef1aF and ef1aR [32] for EF1-α, and COI480F and COI385R [33] or BarS and COIR [32] for COI. PCR products were sequenced by Macrogen Inc. (Santiago, Chile) using the respective 5’-3’ forward primers.

### Sequence editing and multiple alignment

Chromatograms were analyzed using Geneious v.2022.2.2, and low-quality regions were trimmed when necessary. Initially, both marker sequences were subjected to BLASTn (The Basic Local Alignment Search Tool for nucleotides) analysis. In addition, COI sequences were queried through BOLD (The Barcode of Life Data system). Nucleotide sequences were aligned using the Clustal W algorithm in MEGA v.11 [34]. We generated three alignments (A1, A2, and A3) using our own sequences (COI, *n* = 12; EF1-α, *n* = 12) and sequences from GenBank (COI, *n* = 32; EF1-α, *n* = 24, [32]) (Table 2; Supplementary Table 1). Data sets A1 and A2 represent independent alignments of each gene, while data set A3 is a concatenated alignment of both markers, created using Mesquite v.3.81 [35].

**Table 2:** Description of each dataset studied. Molecular markers used, outgroup, total number of sequences and length of the alignment are indicated

Datasets	Molecular marker	Number of sequences	Length (pb)
A1	COI	44	989
A2	EF1-α	36	990
A3	Concatenated	44	1979

### Phylogenetic analysis

Before conducting phylogenetic analyses, a saturation test was performed in DAMBE [36] for each data set to detect potential sequence saturation that could compromise phylogenetic information. This test calculates the saturation index (Iss, Index of substitution saturation) and compares it to the critical value (Iss.c). If Iss < Iss.c, the data is considered unsaturated and suitable for phylogenetic inference; if Iss > Iss.c, saturation is present, which may reduce the accuracy of phylogenetic reconstructions.

Phylogenetic reconstruction was performed using Bayesian inference (BI) and maximum likelihood (ML). The best nucleotide substitution model was selected with the Modeltest tool implemented in IQ-TREE [37], using a codon-partitioning scheme for both genes. BI phylogenies and posterior probabilities (PP) were inferred using MrBayes v.3.2.6 [38] with the Markov Chain-based Monte Carlo (MCMC) method. The analysis was run for 10 million generations, sampling every 1,000 generations to ensure sample independence. A 25% burn-in was applied to discard early trees that had not reached convergence. The parameters were checked with Tracer v.1.7.1 [39]. ML reconstructions were obtained with IQ-TREE v.2.3.6 [40], and node support was evaluated using the ultrafast bootstrap (UFB) method with 100,000 replications [41].

### Molecular species delimitation

Using *cytochrome oxidase I* (COI) gene, three methods were used for species delimitation: one distance-based and two phylogeny-based approaches. These were: (I) ASAP (Assemble Species by Automatic Partitioning), which is a distance-based method that automatically explores distance-based partitions and ranks them using a scoring system (ASAP score) that optimizes within-group cohesion and between-group separation [42]; (II) GMYC (General Mixed Yule Coalescent), which evaluates the branching patterns of an ultrametric tree to differentiate between speciation and coalescence events [43]; and (III) bPTP (Bayesian Poisson Tree Processes), which uses nucleotide substitution counts in a gene tree to infer speciation events [44].

Since GMYC and bPTP require an ultrametric tree, BEAST v.1.10.4 [45] was used to construct the tree, using 10 million generations, sampling every 1,000 generations and a 25% burn-in, all other parameters were left at their default settings. All analyses were conducted on the corresponding web servers: ASAP (https://bioinfo.mnhn.fr/abi/public/asap/), GMYC (https://species.h-its.org/gmyc/), and bPTP (https://species.h-its.org/ptp/), using default parameters in each case.

## Results

### Sample collection and study of external morphology

**Fig. 1:**
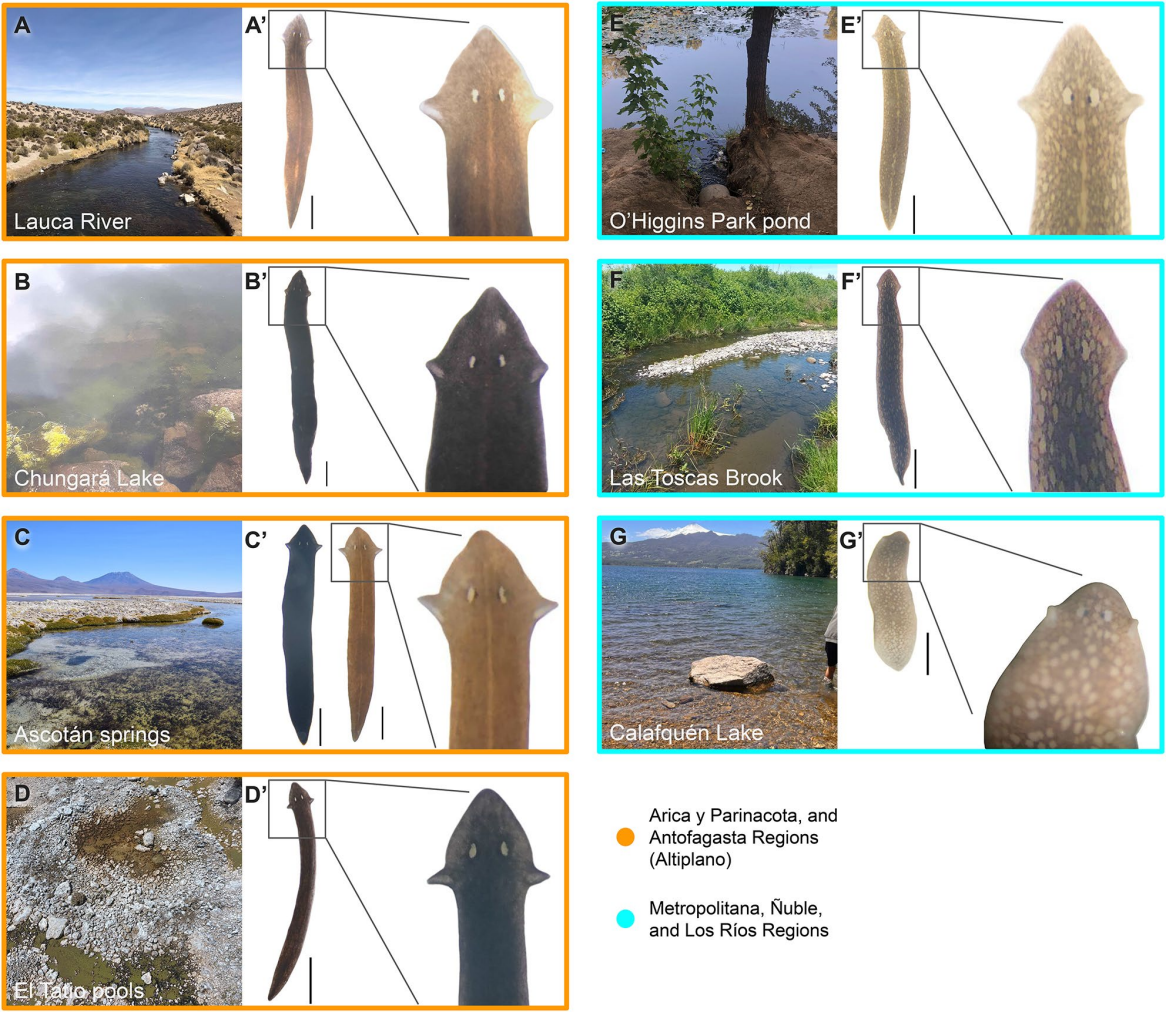
Individuals collected from freshwater habitats. **A**) Lauca River; **B**) Chungará Lake; **C**) Ascotán Salt Pan springs; **D**) El Tatio shallow pools; **E**) O’Higgins Park’s artificial pond; **F**) Las Toscas Brook; **G**) Calafquén Lake. A’ to G’ all living animals in the dorsal view of anterior end, except the one from Calafquén (**G**’), which is ethanol-fixed. Scale bars = 2 mm. A’ to G’ show a Color-coded as in Fig. 2

**Lauca River**. Planarians were found attached to rocks in the Lauca River (Fig. 1A) located at 4,490 m above sea level (m.a.s.l.) in the Lauca National Park, Altiplano. The individuals ranged in size from 1.2 to 2 cm long (n = 6). They exhibited uniform brown pigmentation on both dorsal and ventral sides, and a non-pigmented dorsal stripe running sagittally (Fig. 1A’; Supplementary Fig. 1A). These specimens reproduced sexually, as indicated by a visible gonopore (Supplementary Fig. 1A). Their heads were triangular, with pointed unpigmented auricles, and a pair of ocelli. The pharynx was not analyzed.

**Chungará Lake**. Specimens were collected along the lake’s rocky shore (Fig. 1B), near a belt of macrophytes, located at 4,555 m.a.s.l. in the Lauca National Park, Altiplano. This site yielded a large number of planarians, mostly found on vegetation, alongside other invertebrates such as small snails and copepods. The planarians ranged in size from 1.2 to 2.4 cm long (n = 4). To the naked eye, all specimens appeared completely black. However, under a stereomicroscope, the pigmentation varied across the body but remained in dark gray or black tones, both dorsally and ventrally (Fig. 1B’; Supplementary Fig. 1B). The pharynx was pigmented (Supplementary Fig. 1B). The specimens had triangular heads with pointed unpigmented auricles, and a pair of ocelli. Sexual reproduction could not be affirmed, as we did not find cocoons nor observed a gonopore.

**Ascotán Salt Pan Basin**. Planarians were found attached to rocks in the salt pan springs (Fig. 1C) located at 3,725 m.a.s.l. in the Altiplano. This species reproduces sexually, as evidenced by cocoons found attached to rocks and the presence of a gonopore (Supplementary Fig. 1C). Adult planarians showed a size between 0.8 to 1.8 cm long (n = 15). Two distinct morphotypes were observed (Fig. 1C’), one with black pigmentation (*n* = 6) and another with brown coloration (*n* = 9). Both exhibited homogeneous coloration across their dorsal and ventral surfaces, and a pigmented pharynx (Supplementary Fig. 1C’). Some brown specimens had a lighter midline pigmentation, and intermediate phenotypes. They shared the same morphological traits: triangular heads, pointed unpigmented auricles, and a pair of ocelli. The morphotypes were confirmed by sequencing 18 S (not shown).

**El Tatio Geothermal Field.** Individuals were found in thin shallow pools, no more than 4 cm deep, with muddy bottoms surrounded by siliceous sinter deposits (Fig. 1D). The area was a broad, flat siliceous sinter platform with thin sheets of warm water pools. These waters are considered non-thermophilic, meaning water temperature is under 40°C [46]. This site was located at 4,335 m.a.s.l. in the Altiplano. No planarians were found in a nearby watercourse that runs through the geothermal area, the Salado River. The planarians ranged in size from 1 to 1.3 cm long (n = 9), thinner body shape. Under a stereomicroscope, planarians exhibited dark brown body pigmentation, dorsal and ventral, with dark gray shading on the head (Fig. 1D’; Supplementary Fig. 1D). The pharynx was not analyzed. The head shape was triangular, with long pointed auricles located further back, and a pair of ocelli with very small pigment cups. Sexual reproduction could not be affirmed, as we did not find cocoons nor observed a gonopore.

**O’Higgins Park Artificial Pond**. Planarians were found attached to rocks, alongside other invertebrates such as leeches, hydras, and snails, in the man-made pond (Fig. 1E). This site is located in central Chile at 535 m.a.s.l. The length of these planarians was approximately 1 cm for the individuals examined (n = 4). The planarians had brown pigmentation interspersed with underpigmented areas throughout the dorsal side, giving them a spotted appearance and a variable midline (Fig. 1E’), and homogeneous brown pigmentation on ventral side (Supplementary Fig. 1E). The head was triangular with pointed non-pigmented auricles, and a pair of ocelli. The pharynx was also pigmented (Supplementary Fig. 1E’). This species reproduces sexually, as proven by the presence of a gonopore (Supplementary Fig. 1E) and deposition of cocoons.

**Las Toscas Brook**. Planarians were found under rocks in the stream bed (Fig. 1F). This site is located at 130 m.a.s.l. in central Chile. The population was abundant, observed inhabiting with snails at the collection point. The planarians ranged in size from 1 to 1.5 cm (n = 6). Pigmentation was dark brown with big underpigmented spots along the dorsal and ventral sides, say spotted pattern (Fig. 1F’; Supplementary Fig. 1F). The head was triangular, with triangular auricles and a pair of large ocelli. The pharynx was pigmented (Supplementary Fig. 1F). Cocoons were found adhered to rocks and planarians had gonopores (Supplementary Fig. 1F), indicating sexual reproduction.

**Calafquén Lake.** Planarians were collected from rocky areas at the edge of the lake (Fig. 1G), where the rocks were densely colonized by planarians, often alongside other small invertebrates like insect larvae in the water and mud. This site is located at 205 m.a.s.l. in central Chile. Due to transportation issues, live specimens could not be observed in the lab, and morphological observations were made from samples fixed in ethanol on site. For the same reason, the size of live individuals could not be recorded appropriately. However, it was possible to note that they were shorter in length. The dorsal side had brown pigmentation with a spotted lighter pattern (Fig. 1G’), while the ventral side was pale (Supplementary Fig. 1G). The pharynx preserved some pigments (Supplementary Fig. 1G). The head and auricles could not be clearly evaluated, though they appeared small in preserved animals. Sexual reproduction could not be affirmed, as we did not find cocoons nor observed a gonopore.

### Molecular phylogenetic analysis

BLASTn analyses revealed significant matches with species of the *Girardia* genus from GenBank for all sequences (Supplementary Table 2). Similarly, the BOLD search did not find matches in the database, but the highest similarity percentages also correspond to *Girardia* species. Therefore, we conducted phylogenetic reconstruction using species from the *Girardia* genus. Before analysis, a saturation test confirmed that the sequences were not saturated (Iss < Iss.c in all cases), ensuring that the phylogenetic signal remained robust and suitable for inference.

**Phylogenetic Relationships**. Overall, the phylogenetic inferences from each data set showed consistent topologies with minor differences in clades lacking node support (Supplementary Figs. 2–5), indicating that both nuclear (EF1-α) and mitochondrial (COI) markers provide comparable and complementary phylogenetic information. Maximum likelihood (ML) method displayed fewer nodes with robust support compared to Bayesian inference (BI). The description that follows is based on the tree obtained from data set A3 (Table 2).

**Fig. 2:**
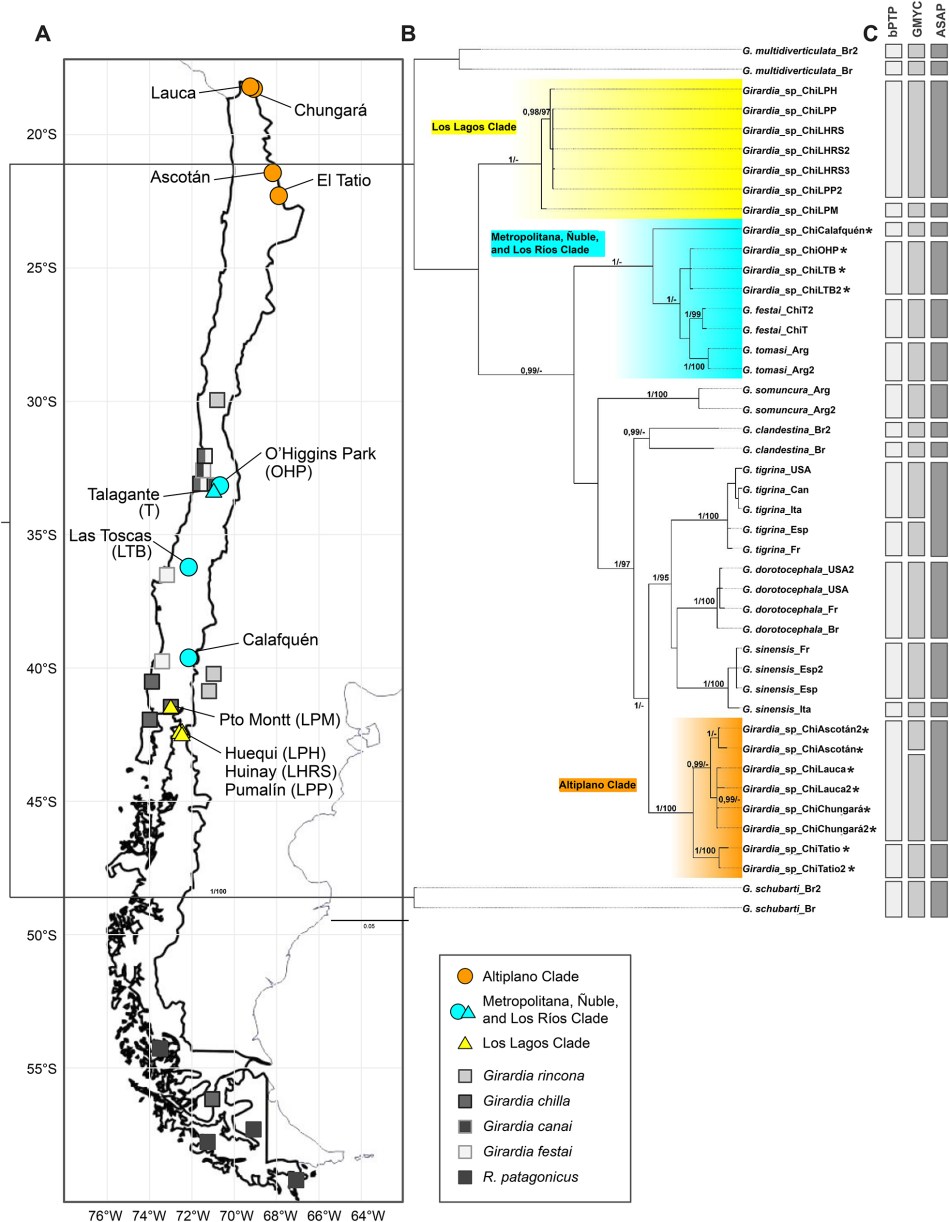
Freshwater planarian biodiversity in Chile. **A**) Map indicating collection sites for freshwater planarians, including those whose DNA sequences were analyzed (circles, this work; triangles, GenBank; squares previously sampled, no DNA sequence available). Dots are color-coded according to the clade each sample belongs to in the phylogenetic tree. **B**) Phylogenetic inference analysis. Clades with at least one Chilean representative are highlighted. In yellow, samples from Los Lagos region; in cyan samples from Metropolitana, Ñuble and Los Ríos regions; and in orange samples from the Altiplano. Node values correspond to PP (posterior probability) and UFB (ultrafast bootstrap) support. Values PP<0.95 or UFB<95 were considered unsupported and are not shown in the figure. Scale: number of substitutions per nucleotide position. Asterisks correspond to sequences from this study. C) Species delimitation analyses. Gray rectangles represent species determined by each delimitation methodology: Bayesian Poisson Tree Processes (bPTP), General Mixed Yule Coalescent (GMYC), and Assemble Species by Automatic Partitioning (ASAP)

As expected, sequences from the same species (including those from GenBank) consistently clustered together (Fig. 2B). Clades formed by *G. schubarti *(Marcus, 1946) [47], *G. multidiverticulata *(de Souza, Morais, Cordeiro & Leal-Zanchet, 2015) [48], *G. tomasi *(Lenguas Francavilla, Negrete, Martínez-Aquino, Damborenea & Brusa, 2021) [49], *G. festai *(Borelli, 1898) [24], *G. somuncura *(Lenguas Francavilla, Negrete, Martínez-Aquino, Damborenea & Brusa, 2021) [49], *G. sinensis *(Chen & Wang, 2015) [50], *G. dorotocephala *(Woodworth, 1897) [51], *G. tigrina *(Girard, 1850) [52], and *G. clandestina *(Sluys & Benitez-Álvarez, 2023) [32] were well supported in both BI and ML methods.

Interestingly, sequences from individuals collected in Chile were distributed across three distinct clades, highlighted in yellow, cyan, and orange in Fig. 2B. All three displayed strong support in Bayesian inference (PP = 1), but only the orange clade also has robust support in maximum likelihood (UFB = 100).

The yellow clade comprises sequences from the Los Lagos Region, including Huequi Peninsula/LPH, Pumalín Park/LPP, Huinay Research Station/LHRS, and Puerto Montt/LPM (Fig. 2A, yellow triangles), all retrieved from GenBank. The cyan clade consists of sequences from the Los Ríos (Calafquén Lake), Metropolitan (O’Higgins Park/OHP), and Ñuble (Las Toscas Brook/LTB) Regions (Fig. 2A, cyan circles), together with *G. festai* from the Mapocho River (Talagante, Chile) and *G. tomasi* from the Valcheta stream (Argentina), both obtained from GenBank. Within this clade, *Girardia*_sp_ChiCalafquén is recovered as sister to all remaining cyan-clade taxa (PP = 1; UFB = 99), while *Girardia*_sp_ChiOHP and *Girardia*_sp_ChiLTB form a well-supported sister group to *G. festai* and *G. tomasi* (PP = 1; UFB = 99). The orange clade comprises all samples from the Chilean Altiplano (3,725 - 4,555 m.a.s.l.), including Ascotán basin, Lauca River, Chungará Lake, and El Tatio (Fig. 2A, orange circles). Within this clade, the El Tatio samples represent the earliest diverging lineage (PP = 1; UFB = 100), while the Ascotán samples form a sister group (PP = 0.99) to the Lauca and Chungará samples, which generate a polytomy.

### Molecular species delimitation

Across all analyses, known species were generally recovered correctly, except for *G. multidiverticulata*, *G. clandestina*, and *G. sinensis*, which were split into multiple units by every method (Fig. 2C), likely due to intraspecific genetic structure.

Our analyses identified six putative species. One lineage corresponds to *Girardia*_sp_Los Lagos, which englobes samples from Huequi Peninsula/LPH, Pumalín Park/LPP, and Huinay Research Station/LHRS, while a second lineage corresponds to *Girardia*_sp_ChiLPM (Puerto Montt), with both units being robustly supported as independent entities across all delimitation analyses. Likewise, *Girardia_*sp_ChiCalafquén was recognized as a separate entity by bPTP, GMYC, and ASAP. The *Girardia_*sp_ChiOHP/LTB lineage was similarly supported by all three approaches, which consistently clustered O’Higgins Park and Las Toscas Brook into a single coherent group. Then, within the Altiplano region, *Girardia_*sp_ChiLauca/Chungará/Ascotán was inferred as a unified species by bPTP and ASAP, whereas GMYC separated Ascotán from Lauca/Chungará but still recovered all three as a well-supported clade. Finally, *Girardia_*sp_ChiTatio was robustly delimited as an independent species by all three methods, highlighting its strong genetic differentiation relative to neighboring Altiplano lineages. *G. festai* (Chile) was excluded from these counts because it is a known species with a formal morphological description.

## Discussion

### Genus and species assignment

The present study uses morphological and molecular evidence to establish the taxonomic identity of the collected specimens. All individuals had triangular heads and pointed auricles (Fig. 1), diagnostic traits at the genus level. Both *Dugesia* and *Girardia* genera display triangular heads, but *Girardia* is distinguished by prominent, pointed auricles, likely considered apomorphic for the group [53]. Specimens from the Altiplano (Lauca River, Chungará Lake, Ascotán Salt Pan, and El Tatio) displayed elongated auricles, resembling those observed in *G. dorotocephala* and *G. bursalacertosa* [54]. Although the pharynx could not be studied in all individuals, a pigmented pharynx, characteristic of *Girardia,* was observed in individuals from Chungará, Ascotán, O’Higgins Park, and Las Toscas (Supplementary Fig. 1). Collectively, these coarse morphological traits support that Chilean specimens analyzed here belong to the genus *Girardia*. Molecular data further support this assignment. BLASTn analyses revealed that the closest matches for all sequences correspond to described species of *Girardia* (Supplementary Table 2). Thus, it is reasonable to conclude that all individuals studied here belong to this genus.

Molecular species delimitation analyses consistently identify multiple genetically independent lineages among the Chilean *Girardia* specimens (Fig. 2C). Across methodologies, the specimens from El Tatio, OHP/LTB, Calafquén and LPM were consistently partitioned as four different lineages, while populations from the Altiplano (Lauca, Chungará, and Ascotán) tended to cluster together. Similarly, individuals from the Los Lagos Region (LPH, LPP, and LHRS) consistently appeared as a well-supported, separate lineage. These results indicate that the analyzed specimens do not represent a single, widespread species, but rather a variety of lineages with geographically restricted distributions. Accordingly, we propose six putative new species: *Girardia* sp. El Tatio, *Girardia* sp. Lauca/Chungará/Ascotán, *Girardia* sp. Calafquén, *Girardia* sp. OHP/LTB, *Girardia* sp. Los Lagos, and *Girardia* sp. LPM.

Confirmation of any of the candidate species delimited here requires internal anatomy data and multilocus or genomic phylogenetic studies. Furthermore, because most *Girardia* species previously described from Chile were established solely on morphological data, lacking molecular information, we cannot reject that some of the lineages identified here correspond to previously described species. In particular, those whose sampling location was nearby, like *G. chilla* and *G. canai* in central Chile (Fig. 2A, squares). None of our molecularly analyzed specimens partitioned with *G. festai* (sequenced by [32]), indicating our samples are at least different from *G. festai*. It is very likely that the Altiplano populations, whose geographic distribution does not overlap with that of any previously reported *Girardia* species, are new unknown species. Future research should focus on sampling from type localities to allow direct genetic comparisons and formal taxonomic evaluation of these potentially new species.

### Phylogenetic relationships of *Girardia* species from sampled areas in Chile

All analyzed specimens belong to *Girardia* genus, endemic to the Americas, and the most diverse genus of freshwater planarians in the Neotropics [8, 54]. In Chile, four of the five previously described species belong to *Girardia*, and our results reinforce its position as the dominant freshwater triclad genus in the country.

Benítez-Álvarez et al. [32] identified two main lineages within *Girardia*: Clades A-B containing species from Brazil, Mexico, and the USA (e.g. *G. schubarti*), and Clades C-R comprising two lineages. A non-resolved lineage containing described and unidentified species from Chile, Brazil, and Mexico (Clades C-J), and a resolved lineage including *Girardia* from the Americas, Europe and others. In our study, *G. schubarti* was the only representative of the first lineage, yet its inclusion allowed us to recover the divergent lineages reported previously. All Chilean specimens analyzed here cluster within the second and third lineages. Within this context, the Chilean *Girardia* display substantial genetic and geographic diversity, as they do not form a monophyletic group, indicating multiple independent evolutionary histories across the country.

Specimens from central Chile show a close phylogenetic affinity to *G. festai* (Chile) and *G. tomasi* (Argentina), forming a well-resolved subclade (Fig. 2B, colored in cyan). Environmental differences among the localities, that is temperate-rainy conditions in Calafquén Lake versus semi-arid transitional climate in Las Toscas Brook [28], could have contributed to diversification within this lineage. The Andes Mountains, acting as a strong geographic barrier with Argentina, may also explain the divergence between *G. tomasi* (Argentina) and Chilean populations. While the Andes constitute a major contemporary geographic barrier between Chile and Argentina, the genetic differentiation observed among Chilean *Girardia* lineages indicates that present-day geographic proximity alone does not fully explain phylogenetic relationships. In particular, the strong divergence of the Los Lagos clade from geographically adjacent populations in the Metropolitan, Ñuble, and Los Ríos Regions contrasts with the relatively close genetic affinity between *G. tomasi* from Argentina and some Chilean lineages. A plausible explanation lies in the complex Pleistocene history of southern Chile. The Los Lagos Region corresponds to the northernmost extent of the Patagonian ice sheets [55], and repeated glacial advances and retreats likely promoted local extinctions followed by recolonization from distinct refugia. In parallel, postglacial hydrological reorganization—including basin capture and the establishment of lake–river chains draining toward the Pacific but originating east of the Andes—may have generated historical connectivity patterns that are decoupled from modern drainage geography. Importantly, Los Lagos also marks the northern onset of the Valdivian temperate rainforest, a distinct ecological zone characterized by high precipitation, stable hydrological regimes, and dense forest cover, which may have contributed to long-term habitat continuity and ecological isolation relative to more seasonal Mediterranean systems to the north. Together, glacial history, hydrological rearrangements, and the presence of the Valdivian Forest ecotype provide a coherent framework to explain the pronounced genetic differentiation of the Los Lagos lineage, while the closer genetic relationship between *G. tomasi* and other Chilean clades may reflect shared ancestry or historical connectivity prior to the establishment of modern geographic and ecological barriers. Nevertheless, the scarcity of molecular data from *Girardia* populations in Argentine Patagonia currently limits a full evaluation of these hypotheses, highlighting the need for expanded sampling east of the Andes to reconstruct the historical phylogeography of the genus.

Remarkably, the Altiplano specimens form a previously unreported monophyletic group within *Girardia* (Fig. 2B, colored in orange). Extreme environmental conditions (aridity, high altitude, intense UV radiation) combined with hydrological isolation may have promoted the diversification of this lineage [56]. Surprisingly, the Altiplano lineage shows greater affinity to North American species (*G. tigrina*, *G. dorotocephala*, *G. sinensis*) than to other South American representatives (*G. festai*, *G. tomasi*, *G. somuncura*, *G. clandestina*, among others). *G. tigrina*, *G. dorotocephala*, and *G. sinensis* are native to North America but introduced to Europe [32, 57–59], where their presence threatens native invertebrate fauna. Regardless, expanded sampling in neighboring Argentina, Peru and Bolivia will be essential for clarifying this unexpected biogeographical pattern.

### Phylogenetic relationships within the Altiplano clade

Phylogenetic analyses revealed three well-defined subgroups within the Altiplano clade: Ascotán, Lauca/Chungará, and El Tatio (Fig. 2B, colored in orange). Geographically, Lauca and Chungará are in close proximity, both located in the Lauca National Park.

Patterns of local diversification in the Altiplano have been reported in *Heleobia* snails where populations from both El Tatio and Ascotán formed a unique lineage (*Heleobia* sp. El Tatio/Ascotán/Lake Blanca) [60, 61]. However, our results indicate that the planarian populations are genetically distinct, likely due to limited dispersal capacity and geographic isolation. Passive dispersal of planarians between these areas is improbable: zoochory, contrary to freshwater snails [61], is rarely reported in planarians [62, 63], and no evidence supports the existence of a paleolake connecting these basins, since the highest known paleolake elevation (3,950 m.a.s.l.) is below that of El Tatio (4,264 m.a.s.l.) [61, 64]. These observations reinforce the hypothesis that the El Tatio population is likely geographically and evolutionarily isolated from that of Ascotán, likely representing a distinct species.

Samples from the Ascotán basin form a sister group to those from Lauca National Park (Lauca River and Chungará Lake). However, only the GMYC analysis recognizes them as different species, while the other two methods group Ascotán, Lauca, and Chungará samples as a single taxon. Nevertheless, similar geographic patterns of diversification have been documented in other Altiplano taxa. For instance, *Biomphalaria* snails exhibit phylogenetic relationships that mirror hydrological basin boundaries, where geographical proximity is consistent with phylogenetic proximity, leaving individuals from different basins (such as Lauca and Ascotan) as independent lineages [65], in the same way, *Orestias* fish from Ascotán and Lauca show two distinct clades [66–68]. In both genera, allopatric speciation is attributed to the isolation of aquatic systems in the region.

Environmental factors, such as water temperature, could also contribute to this species differentiation. In geothermal areas like El Tatio, warmer waters may influence the life cycle and niche of *Girardia* [69, 70], likely contributing to local adaptation and speciation.

Thus, even though molecular delimitation results are partly inconclusive, the combined phylogenetic, geographic, and ecological evidence strongly suggests that the planarian populations of the Ascotán Salt Pan and Lauca National Park represent distinct evolutionary lineages. Further research, incorporating additional molecular markers, morphological data, and broader sampling, will be essential to confirm their taxonomic status and reconstruct the evolutionary history of *Girardia* in this extreme environment.

Finally, as a preliminary integrative study relying on external morphology and single-locus delimitation, species boundaries must be interpreted cautiously. Future research incorporating internal anatomy, multilocus phylogenies, and genomic data will be essential for resolving the taxonomy of these lineages.

## Conclusion

Through sampling efforts in previously unexplored areas, we have documented freshwater planarians in several rivers, lakes, and even extreme environments such as the Altiplano and a geothermal field. This demonstrates a broader presence of planarians in Chile than previously reported. The discovery of these organisms in such environments raises important questions about their potential physiological and ecological adaptations.

Furthermore, Chile’s vast latitudinal range, coupled with the low dispersal capacity of these organisms, suggests a complex evolutionary and biogeographical history, likely involving multiple historical colonizations from diverse regions. In the Altiplano, our results support the hypothesis that species differentiation within this genus is strongly influenced by geographic isolation, shaped by the region’s complex geological and hydrogeological history.

This research provides a foundation for understanding *Girardia* diversity in Chile and highlights the importance of continued exploration of previously unsampled environments to uncover hidden biodiversity in planarians and other taxa. Taken together, these findings begin to reveal an emerging biogeographical pattern for the history of the group in the Americas, underscoring the need for more integrative, multilayered studies that combine morphology, molecular data, and ecological context.

## Electronic Supplementary Material

Below is the link to the electronic supplementary material.

Supplementary Material 1

## Data Availability

The datasets generated and analysed during the current study are available in the GenBank repository. Please find accession numbers in Supplementary Table 1.
