# Supplementary material for: Biodiversity of freshwater planarians (Platyhelminthes, Tricladida, Dugesiidae) in Chile: exploration of unknown species
Source: BMC Ecol Evol. 2026 Mar 28;26:30. doi: 10.1186/s12862-026-02501-3 (PMC13045118; doi:10.1186/s12862-026-02501-3)

Supplementary Material

**Supplementary Table 1.** Specimen, sampling location, COI and EF1-α access codes.

| Specimen | Location | COI access code | EF1-α access code |
| --- | --- | --- | --- |
| *G. schubarti* | Río Grande do Sul, Brasil | OM307079 | OM418648 |
| *G. schubarti* | Río Grande do Sul, Brasil | OM307080 | OM418649 |
| *G. multidiverticulata* | Mato Grosso do Sul, Bonito, Brasil | OM307123 | OM418641 |
| *G. multidiverticulata* | Mato Grosso do Sul, Bodoquena, Brasil | OM307109 | OM418642 |
| *Girardia* sp. (LPH) | Península Huequi, Los Lagos, Chile | OM307119 | OM418637 |
| *Girardia* sp. (LPP) | Parque Pumalin, Los Lagos, Chile | OM307117 | - |
| *Girardia* sp. (LPP) | Parque Pumalin, Los Lagos, Chile | OM307118 | OM418638 |
| *Girardia* sp. (ChiLHRS) | Estación de Investigación Huinay, Los Lagos, Chile | OM307114 | OM418636 |
| *Girardia* sp. (ChiLHRS) | Estación de Investigación Huinay, Los Lagos, Chile | OM307115 | - |
| *Girardia* sp. (ChiLHRS) | Estación de Investigación Huinay, Los Lagos, Chile | OM307116 | - |
| *Girardia* sp. (ChiLPM) | Puerto Montt, Los Lagos, Chile | OM307120 | - |
| *G. festai* (ChiT) | Río Mapocho, Talagante, Chile | OM307167 | OM418644 |
| *G. festai* (ChiT) | Río Mapocho, Talagante, Chile | OM307168 | OM418645 |
| *G. tomasi* | Arroyo Valcheta, Somuncurá, Argentina | MW271863 | - |
| *G. tomasi* | Arroyo Valcheta, Somuncurá, Argentina | MW271864 | - |
| *G. somuncura* | Arroyo Valcheta, Somuncurá, Argentina | MW271865 | - |
| *G. somuncura* | Arroyo Valcheta, Somuncurá, Argentina | MW271866 | - |
| *G. clandestina* | Rio Grande do sul, São Leopoldo, Brasil | OM307085 | OM418690 |
| *G. clandestina* | Rio Grande do sul, Salvador do Sul, Brasil | OM307086 | OM418689 |
| *G. tigrina* | Lago Douglas, Michigan, EE.UU. | OM307163 | OM418672 |
| *G. tigrina* | Lago Ainslie, Nueva Escocia, Canadá | OM307160 | OM418676 |
| *G. tigrina* | Toscana, Italia | OM307101 | OM418683 |
| *G. tigrina* | Río Muga, Cataluña, España | OM307077 | OM418675 |
| *G. tigrina* | Luna, Francia | OM307076 | OM418671 |
| *G. dorotocephala* | Río Lez, Francia | OM307073 | OM349486 |
| *G. dorotocephala* | Río Grande do Sul, Constantina, Brasil | OM307111 | OM349488 |
| *G. dorotocephala* | Carolina Enterprice, EE.UU | OM307136 | OM349491 |
| *G. dorotocephala* | Carolina Enterprice, EE.UU. | OM307138 | OM349502 |
| *G. sinensis* | Río Fluvià, Cataluña, España | OM307089 | OM418669 |
| *G. sinensis* | Río Fluvià, Cataluña, España | OM307090 | OM418664 |
| *G. sinensis* | Putifigari, Sardinia, Italia | OM307099 | OM418655 |
| *G. sinensis* | Montpellier, Francia | OM307121 | OM418656 |
| **This study:** |  |  |  |
| *Girardia_*sp_ChiLauca | Río Lauca, Putre, Región de Arica y Parinacota (-18,193889, -69,273889) | PX939212 | PQ783652 |
| *Girardia_*sp_ChiLauca2 | Río Lauca, Putre, Región de Arica y Parinacota (-18,193889, -69,273889) | PX939213 | PQ783653 |
| *Girardia_*sp_ChiChungará | Lago Chungará, Putre, Región de Arica y Parinacota (-18,235833, -69,181389) | PX939207 | PQ783647 |
| *Girardia_*sp_ChiChungará2 | Lago Chungará, Putre, Región de Arica y Parinacota (-18,235833, -69,181389) | PX939208 | PQ783648 |
| *Girardia_*sp_ChiAscotán | Salar de Ascotán, Ollagüe, Región de Antofagasta (-21,1978611, -68,2573889) | PX939214 | PQ783655 |
| *Girardia_*sp_ChiAscotán2 | Salar de Ascotán, Ollagüe, Región de Antofagasta (-21,1978611, -68,2573889) | PX939215 | PQ783656 |
| *Girardia_*sp_ChiTatio | El Tatio, Calama, Región de Antofagasta (-22,375, -68,018056) | PX939216 | PQ783657 |
| *Girardia_*sp_ChiTatio2 | El Tatio, Calama, Región de Antofagasta (-22,375, -68,018056) | PX939217 | PQ783658 |
| *Girardia_*sp_ChiOHP | Laguna Artificial Parque O'Higgins, Santiago, Región Metropolitana (-33,4688611, -70,6609722) | PX939211 | PQ783651 |
| *Girardia_*sp_ChiLTB | Estero Las Toscas, Chillán, Región de Ñuble (-36,600268, -72,0723557) | PX939209 | PQ783649 |
| *Girardia_*sp_ChiLTB2 | Estero Las Toscas, Chillán, Región de Ñuble (-36,600268, -72,0723557) | PX939210 | PQ783650 |
| *Girardia_*sp_ChiCalafquén | Lago Calafquén, Puerto Curihue, Región de Los Ríos (-39,5445499, -72,1029345) | PX939206 | PQ783654 |

**Supplementary Table 2.** BLASTn results. Best hit for each sample.

|  | ***Identity (%)*** | ***Query cover (%)*** | ***Species*** |
| --- | --- | --- | --- |
| *Girardia*_sp_ChiLauca | 90.80 | 99 | *Girardia dorotocephala* |
| *Girardia*_sp_ChiLauca2 | 91.69 | 100 | *Girardia* sp. |
| *Girardia*_sp_ChiChungará | 91.76 | 100 | *Girardia dorotocephala* |
| *Girardia*_sp_ChiChungará2 | 91.00 | 100 | *Girardia dorotocephala* |
| *Girardia*_sp_ChiAscotán | 90.75 | 100 | *Girardia dorotocephala* |
| *Girardia*_sp_ChiAscotán2 | 89.92 | 100 | *Girardia dorotocephala* |
| *Girardia*_sp_ChiTatio | 90.75 | 100 | *Girardia dorotocephala* |
| *Girardia*_sp_ChiTatio2 | 89.92 | 100 | *Girardia dorotocephala* |
| *Girardia*_sp_ChiOHP | 96.88 | 85 | *Girardia* sp. |
| *Girardia*_sp_ChiLTB | 96.72 | 98 | *Girardia* sp. |
| *Girardia*_sp_ChiLTB2 | 96.61 | 95 | *Girardia* sp. |
| *Girardia*_sp_ChiCalafquén | 89.16 | 100 | *Girardia* sp. |

**Supplementary Figure 1.** Live animals, except D and G, in ventral view. Abbreviations used: go=gonopore, m=mouth, ph=pharynx.


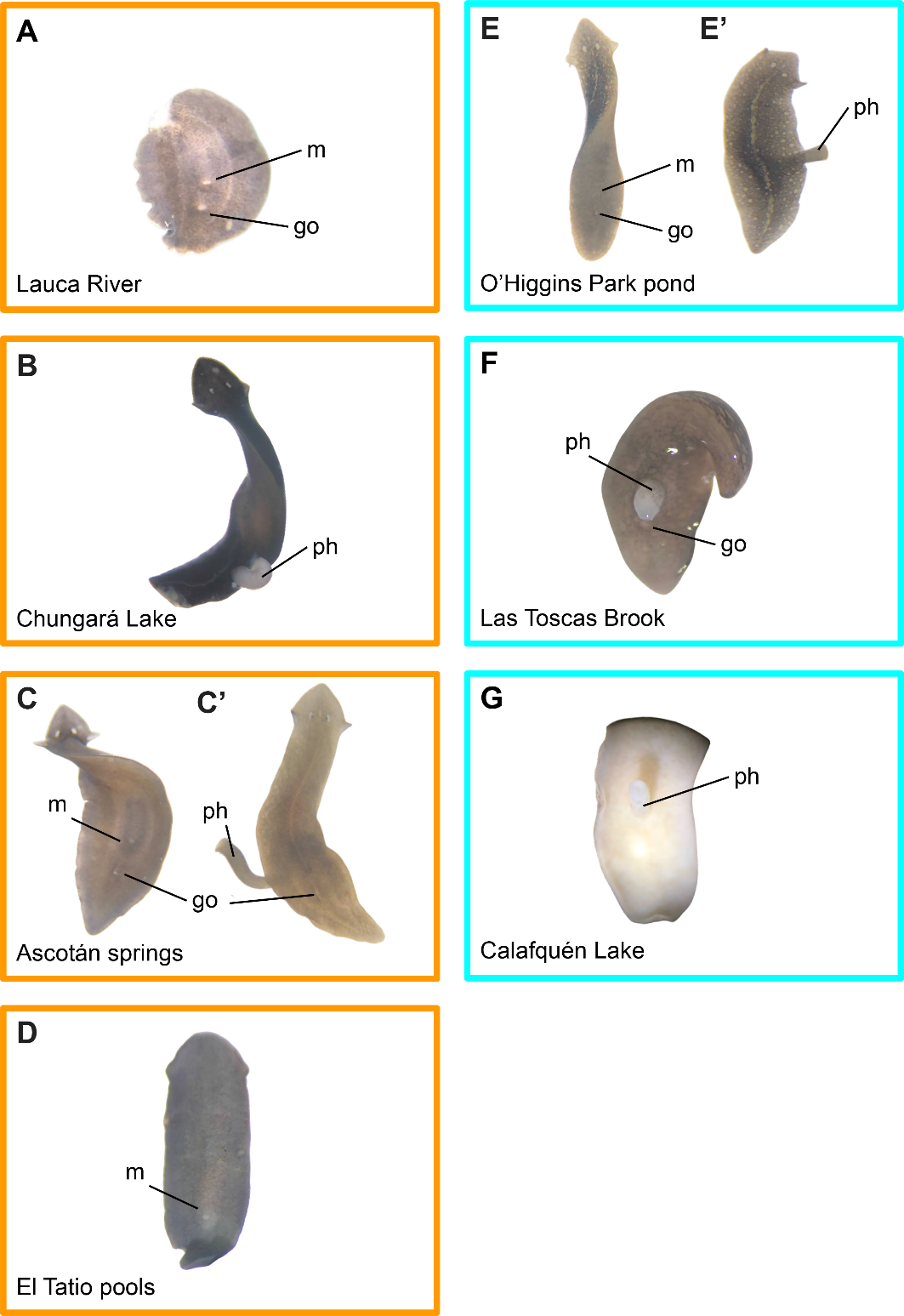


**Supplementary Figure 2.** Bayesian inference performed from the data set A1 (*COI*). Value at the nodes corresponds to the posterior probability. Scale: number of substitutions per nucleotide position.


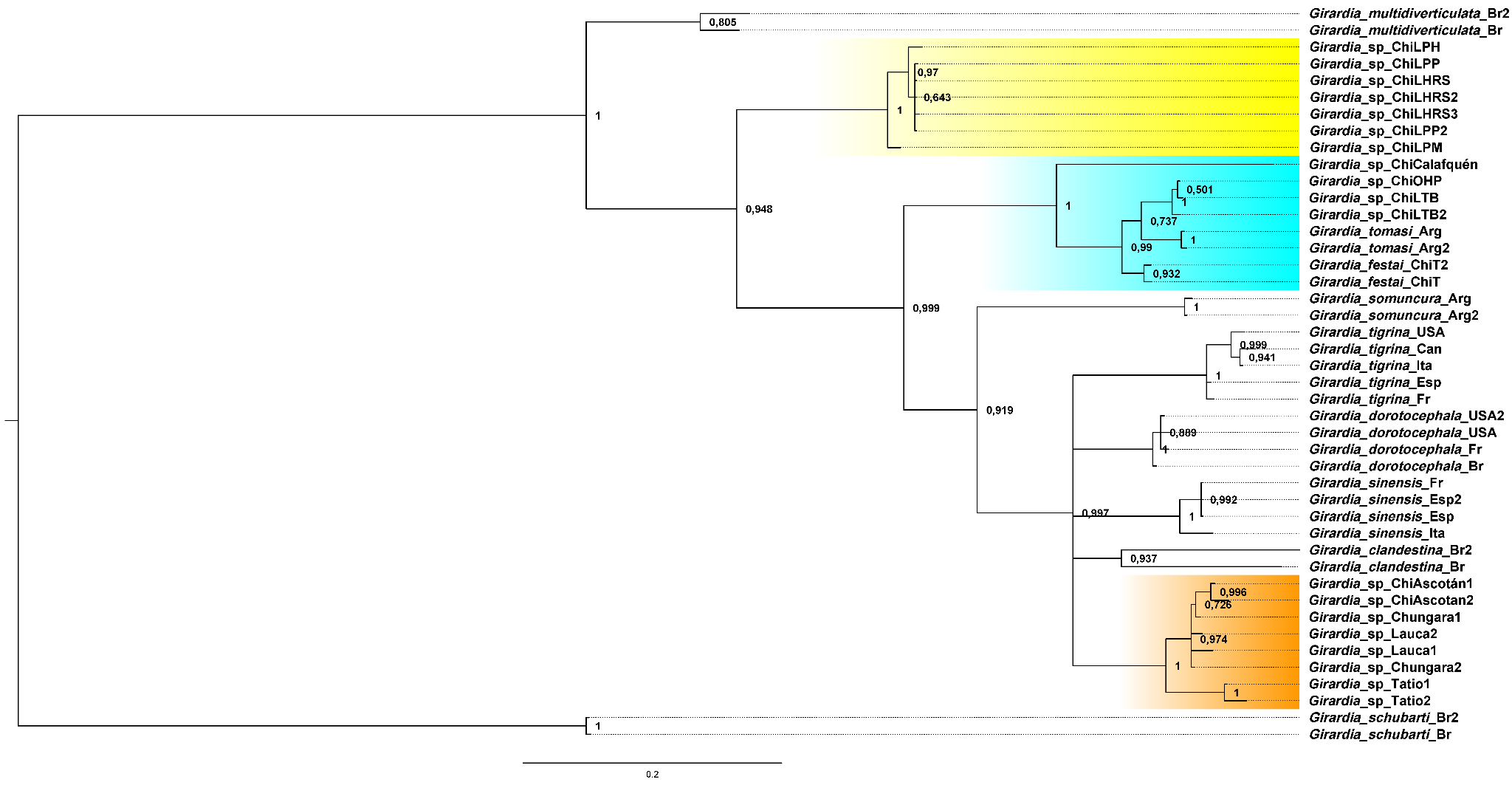


**Supplementary Figure 3.** Bayesian inference performed from the data set A2 (*EF1-α*). Value at the nodes corresponds to the posterior probability. Scale: number of substitutions per nucleotide position.


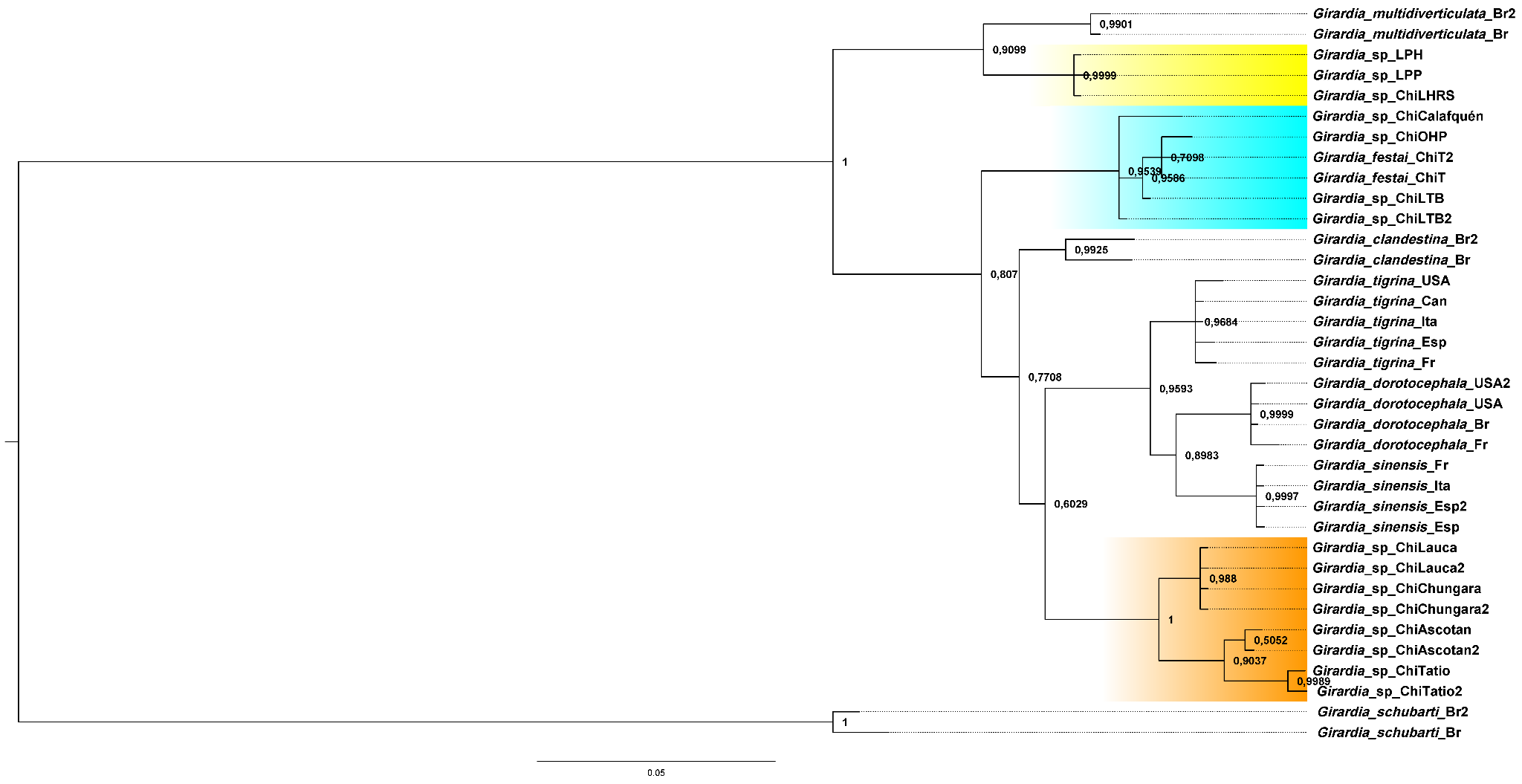


**Supplementary Figure 4.** Maximum Likelihood reconstruction performed from the data set A1 (*COI*). Value at the nodes corresponds to the ultrafast bootstrap support. Scale: number of substitutions per nucleotide position.


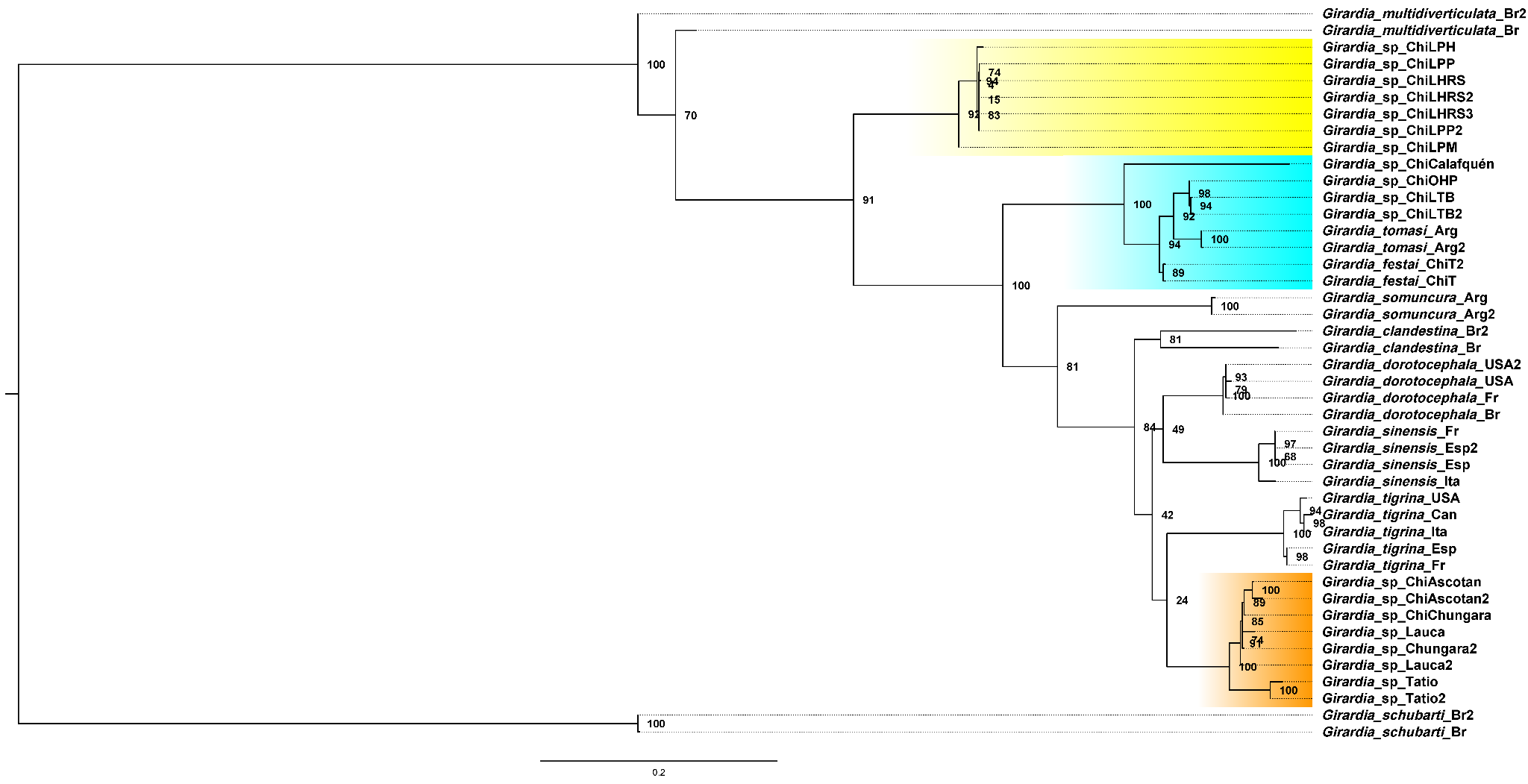


**Supplementary Figure 5.** Maximum Likelihood reconstruction performed from the data set A2 (*EF1-α*). Value at the nodes corresponds to the ultrafast bootstrap support. Scale: number of substitutions per nucleotide position.


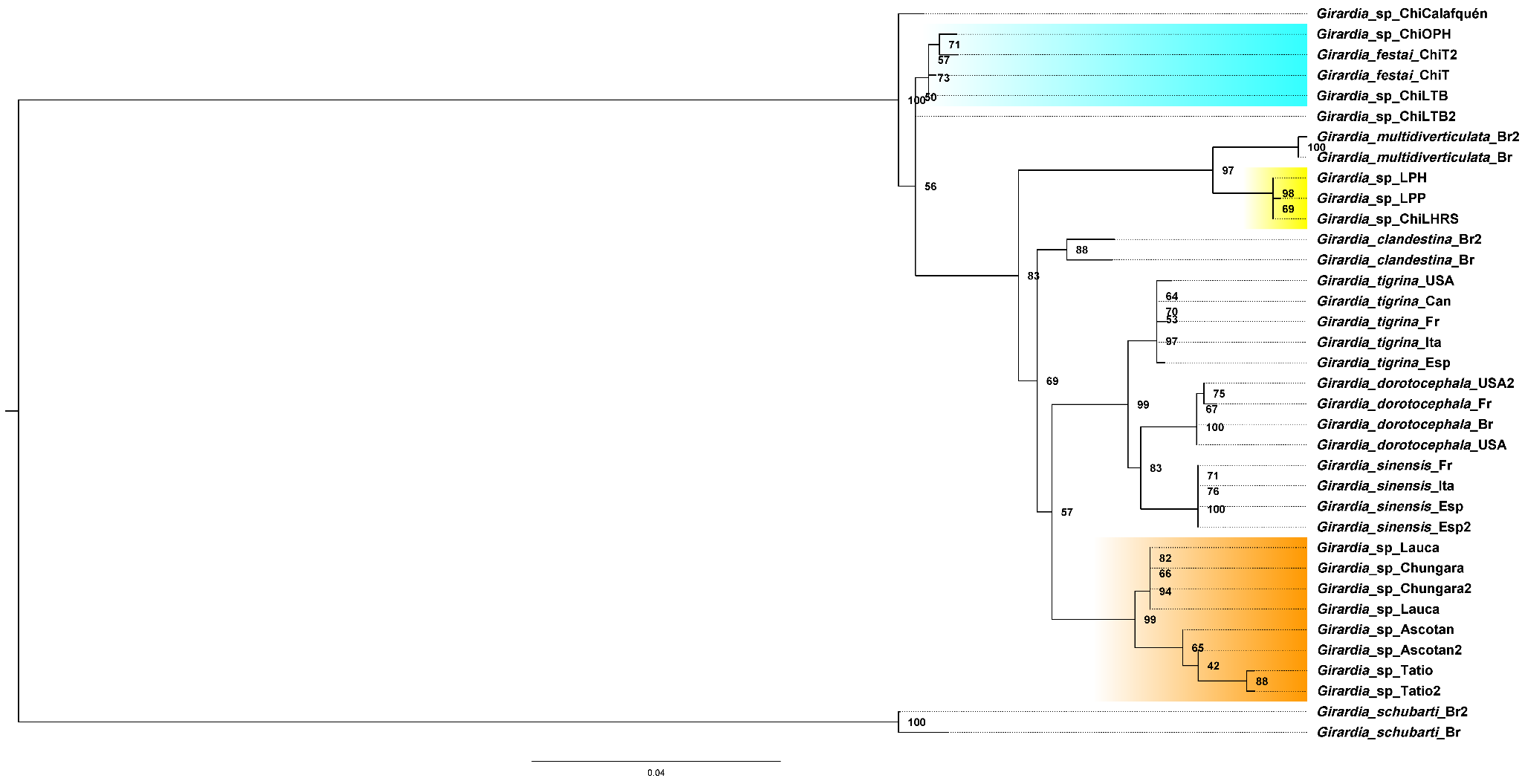

Supplement: Supplementary file 1 — Supplementary Material 1 [file 12862_2026_2501_MOESM1_ESM.docx]
